# Supplementary material for: Are carbon emissions trading and green financial instruments synergistic? -Comprehensive quantitative research based on content analysis
Source: PLoS One. 2024 Mar 7;19(3):e0298601. doi: 10.1371/journal.pone.0298601 (PMC10919601; doi:10.1371/journal.pone.0298601)
Supplement: S1 Data — (ZIP) [file pone.0298601.s001.zip › Data/地级市Z_F工作报告.zip!/║■▒▒/╛ú╓▌╩╨/└┤╫╘╣1⁄2╓┌║┼í╛┬φ┐╦╩2╛▌═°í┐_2018.docx]

各位代表：

现在，我代表市人民政府向大会报告工作，请予审议，并请各位政协委员和列席人员提出意见。

一、2017年工作回顾

过去一年，是新一届政府接续振兴重担的开局年，也是我市勇当多极排头兵的起步年。在省委、省政府和市委的领导下，在市人大和市政协的监督支持下，我们团结和依靠全市人民，深入贯彻党的十九大精神，以习近平新时代中国特色社会主义思想为指导，有效应对经济下行压力，统筹抓好改革发展稳定各项工作，保持了经济社会平稳健康发展。

一年来，我们综合施策、精准发力，经济运行持续回升向好。全年实现地区生产总值1922.2亿元、增长7.5%，固定资产投资总额2232.3亿元、增长11.5%，规模以上工业增加值增长8.2%，社会消费品零售总额1168.6亿元、增长10.6%，地方一般公共预算收入122.5亿元、增长8.7%，城乡居民人均可支配收入分别达到29973元、15962元，增长8.3%、8.5%。

一年来，我们狠抓项目、扩大投资，推动了一批重大项目落地见效。荆州民用机场正式获批开工，荆荆高铁、城北快速启动建设，华电江陵电厂一期、荣成再生科技一期、德尔福线束、能特科技医药中间体、金彭车业等一批工业项目建成投产。

一年来，我们注重民生、强化督办，打响了“三大攻坚战”，成功创建了国家卫生城市、省级文明城市，捧回了全国综治最高奖“长安杯”，完成了全省最重的棚改年度任务，圆满举办了首届荆州国际马拉松，获批省第二届园林博览会举办城市。

荆州的每一点发展变化，都是全市人民凝心聚力、真抓实干的结果。实践反复证明：“路虽远，行则将至；事虽难，干则必成。”荆州人善于谋势、敢于克难、能成大事，只要我们团结一心、埋头苦干，就一定能汇聚强大发展合力，开创加快振兴崭新局面。

回顾过去一年，我们主要做了以下工作。

一是着力抓改革，发展动能更加强劲。

供给侧结构性改革不断深化。“三去一降一补”深入推进。化解煤炭行业过剩产能24万吨，水泥和玻璃行业产能置换达标。信贷结构进一步优化，非法集资有效遏制，金融机构不良贷款余额、比率双下降。房地产去库存周期由13.8个月下降至7个月。落实各项减税降费政策，直接减轻企业税费负担15亿元以上。实施8大类补短板重点工程，基础设施、生态环境、科技创新等薄弱领域得到加强。

“放管服”改革成效显著。新增市场主体6.2万户、同比增长14%。在全省率先推出“33证合一”。相对集中行政许可权改革加快推进，委托下放市级行政审批事项35项，行政服务中心278个审批事项中198个实现“一次办好”，项目建设行政审批时限由156天压缩到60天以内。荆州开发区深化“一枚印章管审批，一支队伍管执法”，工业项目基本实现“零收费、零审批”。部门监管职能进一步规范，“双随机一公开”全覆盖。完善国地税合作“荆州模式”，成功创建全国百佳国税地税合作示范区。

重点改革强力推进。实施农村集体产权制度改革试点，完成农村宅基地和集体建设用地确权登记。深化投融资体制改革，新落地PPP项目总投资116亿元，发行企业债券48.9亿元，新增“四板”挂牌企业431家，量、质排名全省第一。完成23家行政事业单位经营性资产管理改革。城管体制改革启动实施。广播电视台实现整合。国有林场改革和盐业监管体制改革基本完成。医药卫生体制、机关事业单位养老保险、户籍制度、供销社等领域改革取得新进展。

二是着力抓产业，经济实力有效提升。

工业竞争力增强。净增规模以上工业企业45家、达到1283家。税收过千万工业企业净增28家、达到84家。新增高新技术企业70家、达到173家。信息化、工业化融合试点示范企业达到140家，其中省级37家。农产品加工、纺织服装、医药化工、轻工建材、装备制造、电子信息六大产业集群加速壮大，产值同比分别增长6%、9.3%、15.4%、23.2%、15.2%和2.5倍。湖北华讯方舟获国家专利金奖。沙隆达与“全球农化十强”以色列安道麦成功合并。凯乐科技联合15家企业建设总部基地，规划打造量子通信千亿产业。

服务业态势良好。商贸流通业规模扩大，新增“限上”法人企业100家。电子商务发展迅猛，网络零售额突破200亿元、增长50%。全域旅游加速推进，旅游综合收入260亿元、增长30%。华夏历史文化科技园完成投资18亿元，荆州海洋世界一期建成开园。光大银行、渤海银行落户营业。全市金融机构新增贷款余额181亿元、同比多增66亿元，社会融资规模279亿元、同比增长161.2%。外贸出口总额12.2亿美元、增长23.9%。

农业基础不断巩固。新建高标准农田53万亩。粮食产量增长4.7%。再生稻发展经验全国推广，稻田综合种养面积233万亩。农产品加工业产值1862亿元，规模以上农产品加工企业新增26家、达到563家，新型经营主体、省级以上农业产业化龙头企业数量居全省前列。新增“三品一标”产品47个、达到366个。松滋市获评“全国畜牧业绿色发展示范县”，监利县被授予“中国小龙虾第一县”，洪湖市荣获“中国名蟹第一市”，石首“笔架鱼肚”代表湖北亮相世界地理标志大会，公安县建成长江中下游最大葡萄产区，江陵县入选全省优质粮食工程示范县。荆江大堤综合整治、荆南四河堤防加固等重大水利项目基本完成，洪湖蓄滞洪区东分块工程完成投资19.8亿元。

三是着力抓保护，生态环境持续好转。

长江大保护扎实推进。荆江航道整治工程竣工，三大国家级保护区生态环境明显改善。中央环保督察反馈问题整改完成序时进度。78个项目纳入国开行长江大保护融资项目库。非法码头整治强力推进，拆除、取缔长江干线码头泊位353处，清退岸线55公里。非法采砂有效遏制。清理环保违法违规企业1360个，关闭落后产能“十五小”企业71家、污染严重“十小”企业和非法作坊116家。国家森林城市创建全面推进，完成造林绿化62.2万亩，森林覆盖率提高3个百分点。

水土污染防治成效明显。建成城区污水管网38.4公里。建立市县乡三级河湖库长制，在全省率先启动湖泊保护“一湖一法”，洪湖、长湖围网全部拆除。退垸还湖启动实施。湿地保护面积达到19万公顷，国家级、省级湿地公园达到11个，自然湿地面积达到71.2万公顷。市县两级饮用水保护区划分全部完成，14个地表水考核断面达标率提高到79%，集中式饮用水水源地水质达标率100%。实施土壤污染防治行动，基本农田区、蔬菜种植区、饮用水源地均保持较清洁以上等级。

空气质量进一步改善。开展秸秆禁烧、清洁能源替代、扬尘管控和机动车尾气专项治理，秸秆综合利用率达到96%，黄标车和市县中心城区燃煤锅炉全部淘汰，建筑施工场地防尘“六化”率达100%。中心城区PM10、PM2.5浓度同比分别下降7%、6.7%，空气优良天数达到273天、较上年增加36天。

四是着力抓统筹，城乡面貌日益改善。

城市建设步伐加快。“一城三区、一区多园”市级统筹体制初步建立，中心城区差异化发展格局加快形成。市中心医院、市一医院门诊大楼等项目进展顺利，市图书馆新馆建成开放，荆州中学、沙市中学新校区建成使用，荆州职业技术学院改扩建一期项目完成。护城河清淤、古城墙修缮等世行贷款项目开工建设。棚户区改造开工71582套，其中中心城区61376套，均居全省第一。“荆楚云”上线运行。“五城同创”深入推进。被确定为全国第二批城市设计试点城市、第二批“中德生态示范城”。公安县获评国家卫生县城，石首、监利获评国家园林城市。

城乡交通网络加快完善。蒙华铁路荆州长江公铁大桥主体工程完工。沙公高速、石首长江大桥及接线、洪湖至嘉鱼长江大桥等项目快速推进，洪湖至赤壁长江大桥正式开工。洪监高速复工。撤除荆监一级公路收费站。207国道城区段完成大修，318国道城区段改线工程顺利实施。新增普通一二级公路175公里。“四好农村路”建设取得实效，新建农村公路800公里。完成生命安全防护“455”工程2800公里。

美丽乡村建设成效初显。省级试点村达到43个，建成省级特色小镇5个，洈水镇入选全国特色小镇。特色农家乐、民俗特色村、休闲农业示范园快速发展。82个新建乡镇生活污水处理厂全面开工，荆州区、松滋市农村生活垃圾处理通过省级年度验收。农村电网改造完成投资10.6亿元。1125个村实现光纤网络全覆盖，4G无线信号实现村级全覆盖。

五是着力抓民生，群众获得感不断增强。

精准脱贫务实有效。“六大攻坚行动”深入实施。统筹22.9亿元用于精准扶贫。实施各类产业扶贫项目771个，带动贫困户10473户。发放金融扶贫小额贷款5.6亿元。建成村级光伏电站248座。健康扶贫体系初步建立。60个贫困村、42878人减贫任务如期完成。

社会保障更加有力。新增城镇就业8.5万人，城镇失业人员再就业3万人，就业困难人员实现就业1.5万人。发放小额担保贷款新增2.5亿元，扶持创业5473人。五项社会保险参保达到768.5万人次，征收社会保险费60亿元。发放公积金个人贷款19亿元。城乡低保、五保供养全面提标。新建城乡社区居家养老服务中心138个，民办养老机构增至35家。发放残疾人“两项补贴”6617万元。

社会事业加快发展。为民办实事基本完成。申报科技成果转化项目80项，发明专利授权量231件。8个县市区通过县域义务教育均衡发展国家督导评估认定。高中教育质量稳步提升。职业教育整合力度加大。“文化三市”创建加快。大型原创汉剧《优孟衣冠》上演武汉、走向广州深圳，歌剧《有爱才有家》入选文化部民族歌剧重点扶持工程。“荆楚文物珍品展”随荆州舰出访20个国家。青少年体育、群众体育走在全省前列，洪湖市、石首市、公安县荣获全国群众体育先进县。城乡居民医保整合完成。全面两孩政策实施效果明显，新生儿二孩占比52.1%。荆州区、沙市区、江陵县、荆州开发区血吸虫病传播阻断达标。沙市区通过全省首批食品安全城市试点验收。防范化解重大风险攻坚战成效明显。拖欠农民工工资问题有效治理。解决了一大批信访积案。进京非正常上访下降42.8%。安全专项整治进一步深化。“平安荆州”创建力度加大，刑事案件、治安案件同比分别下降11.5%、12.6%，社会大局保持和谐稳定。

国防动员、国防教育、国家安全、人民防空、民兵预备役工作得到加强。应急管理、防震减灾、对口支援、台湾事务、外事侨务、民族宗教等工作取得新成绩，机关事务管理、统计、物价、农垦、移民、烟草、慈善、气象、档案、社科、史志、保密和无线电等工作取得新进步。

各位代表！抓落实是全部政府工作的精髓。一年来，我们坚持狠抓政府“大督查”体系建设，紧扣“事前、事中、事后”三环节，突出“时间表、责任人、督办、问责”四要素，推行“末位表态、后进发言”制，采用挂图作战、微信群调度等方式，晒业绩、比位次、督进度，努力推动各项工作落到实处。始终坚持依法行政，落实政府法律顾问制度、行政执法主体资格制度，办理本级人大代表建议167件、政协提案310件。始终坚持抓党建转作风，深入贯彻落实中央八项规定精神，扎实开展“人情风”“庸懒症”“圈子文化”专项整治，切实加强效能监察和审计监督，政府行政效能明显提升。

各位代表！政府工作的每一点进步，都离不开省委、省政府和市委的正确领导、科学决策，离不开市人大、市政协的有效监督、鼎力支持，离不开社会各界的关心帮助、热情参与。在此，我代表市人民政府，向全市广大干部群众，向驻荆部队、武警官兵，向各民主党派、工商联、无党派人士、各人民团体和社会各界人士，致以崇高的敬意！向所有关心、支持、参与荆州改革开放和现代化建设的中外朋友，表示衷心的感谢！

各位代表！在肯定成绩的同时，我们也清醒地认识到，我市经济社会发展中仍然存在一些突出困难和问题。一是经济总量不大、人均水平不高，经济运行抗风险能力较弱，尤其是工业发展不够、投入严重不足，缺乏大项目支撑，成为荆州经济发展的最突出短板。去年地区生产总值、规模以上工业增加值等重要经济指标没有达到预期。二是综合交通不完善，高速公路、铁路人均里程少，过江通道不足，交通问题仍然是制约荆州发展的瓶颈。三是污染治理与生态修复任重道远。环保历史欠账多，投入缺口大，农业面源污染量大面广，水环境修复任务尤为艰巨。四是发展环境有待进一步优化，“放管服”改革还不全面、不彻底，群众和市场主体获得感不强，企业融资难、融资贵等问题尚未得到有效解决。民生保障还存在较大差距。一些部门和工作人员进取意识、责任意识、服务意识缺乏，“庸懒症”问题突出，等等。针对这些问题，我们将认真面对、有效施策，切实加以解决。

二、2018年重点工作

各位代表！今年是贯彻党的十九大精神的开局之年，是决胜全面建成小康社会、实施“十三五”规划承上启下的关键一年。

从宏观大势看，全球主要经济体逐渐复苏，中国经济巨轮行稳致远，长江经济带、长江中游城市群、中部地区崛起、洞庭湖生态经济区等战略惠及荆州，利于我们应势而谋；从区域态势看，湖北经济稳中向好，省委、省政府明确支持荆州打造全省多极发展排头兵，需要我们顺势而动；从自身趋势看，“一城三区、一区多园”顺利实施，综合交通快速发展，一批大项目、好项目加快推进，催促我们乘势而为。更为重要的是，全市上下聚力发展的气场日益强大，奋发赶超的斗志已经凝成，更加坚定了我们抢抓机遇、担当作为的信心和决心。

今年政府工作的指导思想是：高举习近平新时代中国特色社会主义思想伟大旗帜，深入学习宣传贯彻党的十九大精神，认真落实中央经济工作会议、省委十一届二次全会、市委五届五次全会精神，坚持新发展理念，按照高质量发展的要求，统筹推进“五位一体”总体布局和协调推进“四个全面”战略布局，以供给侧结构性改革为主线，坚决打赢“三大攻坚战”，共抓大保护，厚植大开放，构筑大交通，培育大产业，建设大城市，奋力开创“勇当多极排头兵、实现荆州大振兴”新局面。

今年经济社会发展的主要预期目标是：地区生产总值增长7.8%左右，固定资产投资增长13%，规模以上工业增加值增长8.5%，社会消费品零售总额增长11%，地方一般公共预算收入增长8.5%；居民人均可支配收入增长高于经济增速，城镇登记失业率控制在4.5%以内，完成省下达的节能减排任务。

（一）做大做强产业支撑。以习近平新时代中国特色社会主义经济思想指导经济工作，深入推进“一城三区、一区多园”功能分区、产业布局，着力提升产业配套能力和综合竞争力，加快构建现代化经济体系。

推动工业增量升级。工业是调结构、转方式的主战场，更是稳增长、促发展的主力军。以荆州开发区为主平台引领新型工业化。按照“一区多园”管理机制，聚焦主导产业，完善基础设施和功能配套，引导项目向同类园区聚集，加快建设新能源汽车及装备智能制造产业园、军民融合暨光通讯电子信息产业园等专业园区。坚定不移壮大产业。加快推进中小企业成长，培育科技“小巨人”，促进个转企、新进规、规改股、股上市，新增规模以上工业企业50家。对接中国制造2025行动纲要，实施“千企千亿技改工程”，抓好传统产业转型升级，三年内培育3家年销售收入过100亿元企业、100家细分领域潜力领军企业。做大做强战略性新兴产业。以汽车零部件产业集群为支撑，加快实施汽车产业发展规划，积极发展新能源汽车产业。加强运行调度。筹建荆州工业云，注重工业企业运行监测、分析与研判。尊重、关爱企业家，保护、弘扬企业家精神。落实“一企一策”点对点精准服务，提高要素保障水平。

推动农业增效提质。坚持质量兴农、绿色兴农，加快农业供给侧结构性改革和农业现代化建设，为乡村振兴注入发展新动能。以荆州高新区为示范引领现代农业发展。按照“产学研结合，一二三产融合”的思路，完善基础设施，创新体制机制，建设江汉平原农业科技创新中心、湖北省水产产业技术研究院等农业创新平台；深化与高校、科研单位合作，引进一批院士专家工作站、研发中心和农产品加工行业龙头企业，创建以农业为特色的国家级高新区。深入推进农业供给侧结构性改革。开展“农业质量年”活动，新建高标准农田40万亩，重点发展水稻、水产，优化推广稻渔共作和再生稻等绿色模式。全面禁止高毒农药，推广有机肥，探索农业废弃物集并、回收、转化利用，创建国家农业可持续发展试验示范区、省级特色农产品优势区，推进粮食生产功能区、重要农产品生产保护区建设。加快土地流转步伐，规范流转秩序，流转率达到70%。加快培育农业新型经营主体，发展各类股份合作社。深入推进农产品加工业倍增计划，新增规模以上农产品加工企业30家。做强“荆楚味道”公共品牌。加强农田水利建设。加快建设洪湖蓄滞洪区东分块、长湖洪湖湖堤加固等工程。推进高效节水灌溉、大中型灌区及基本农田水利建设。

推动第三产业突破性发展。以纪南文旅区为重点引爆荆州文化旅游业。将文化旅游业培育成为荆州未来的核心竞争力，按照“中国品牌、湖北代表”目标，推动纪南文旅区做大做强。全力推进荆州古城5A创建。丰富旅游产品供给，优化旅游线路，提升旅游景点、景区服务水平，改善景区环境。繁荣发展商贸物流业。新增“限上”商贸企业100家。加快建设区域性流通节点城市。举办荆楚美食节，擦亮荆州老字号，进一步活跃消费市场。加强农产品城乡终端市场建设，升级改造农产品批发市场和物流中心，支持建设一批综合物流园项目。积极组织相关企业拓展海外市场。大力发展现代金融业。打好防范化解金融风险攻坚战。引导银行类金融机构加大对实体经济特别是中小微企业的信贷投放，全年新增贷款200亿元。加快资本市场建设，新增“新三板”挂牌10家,引导“四板”挂牌企业扩大直接融资。开展金融招商，积极引进各类银行机构。用足用活产业基金，逐步完善基金品类。统筹调度政府性资金，支持政府担保平台做大做优。支持沙市区建设省级现代服务业试点示范区。

狠抓项目建设和招商引资。坚持把每一项发展任务落脚到项目载体上，充分发挥投资对优化供给结构的关键作用。紧紧扭住项目建设“牛鼻子”，坚持领导干部领衔重大项目，落实固定资产投资调度、重点项目联系服务制度，继续开展“重大项目开工月”活动，推动各类项目落地生根、开花结果。坚持“三资”共抓，突出招商引资“一号工程”地位，围绕产业链和产业集群，瞄准世界500强、中国500强和行业领军企业，开展园区招商、专班招商、回归招商、重资产招商和基金招商，引进2个50亿元以上、350个亿元以上工业项目。以开工为核心环节，加强督查考核，确保招商引资实效。

（二）深入推进改革开放创新。今年是改革开放40周年，必须坚持以改革的思路破解发展中的难题，以改革的勇气加速后发赶超。

深化重点领域改革。抓好供给侧结构性改革，继续推进“三去一降一补”，注重在“破、立、降”上下功夫。全面推行“一窗受理”“一网覆盖”“一次办好”服务，进一步清理部门行政审批权，推动“放管服”改革成效第三方评估全覆盖。深入推进工业项目建设“零审批”改革。创新“多评合一”，完善投资项目联审联办平台。升级“互联网+政务服务”，推进政府系统信息共享，加快建设掌上政务平台，推动群众办事“网购化”。深化财政管理改革，全面推行绩效管理，以县市区为单位推进项目统筹、资金整合。做大做强荆州城投、荆州海投和县市投融资平台，创新实施准公益性项目融资模式。落实“三权分置”，推进农村集体产权制度改革，完成农村土地承包经营权确权登记颁证。完成国有农场办社会职能改革。推进混合所有制改革试点和国有资本经营预算全覆盖。深化医疗卫生体制改革。加强社会信用体系建设，创建全国社会信用示范城市。

努力扩大对外开放。把中心城区打造成对外开放的主平台和承接产业转移的主导区。加快荆州水运口岸对外开放，重点推进国际贸易“单一窗口”建设，申报设立海关特殊监管区（场所）和专业特色口岸。对接湖北自由贸易试验区，探索复制投资自由化、金融国际化、贸易便利化等制度。继续壮大出口支柱产业和重点企业，提升荆州制造“走出去”能力。加快新型业态建设，引进大型外贸综合服务示范企业。大力发展跨境电商，提升荆州出口品牌，争创国家级汽车零部件和纺织品出口示范基地。高标准规划建设临空临铁临港经济区。

强化科技创新支撑作用。紧紧抓住创新“第一动力”、把握转型升级“主攻方向”，以科技创新引领传统产业优化升级。创建国家创新型试点城市，加大政策支持力度，提升研发经费支出占生产总值比重、高新技术产业占规模以上工业比重，新增高新技术企业30家。强化企业创新主体地位，构建以企业为主体、市场为导向、产学研相结合的技术创新体系，促进更多科技项目成果转化、落地。加快创新平台建设，引导现有孵化器建成共享性专业技术服务平台，新增在孵企业、创客150家。加大对农业类科研院所、特色“种养加”示范基地支持力度。实施“我选湖北·凤鸣荆州”人才计划，鼓励大众创业、万众创新，积极打造人才流入城市，形成千帆竞发、百舸争流的创新发展新局面。

（三）加强生态文明建设。像对待生命一样对待生态环境，狠抓长江大保护，建设美丽荆州。

突出水环境治理。深入实施“水十条”，认真落实河湖库长制，系统推进“四水共治”。开工建设荆江沙市段和环长湖生态修复与保护PPP项目。开展“三年行动”，统筹推进四湖总干渠综合整治。落实主要水体水质报告公开制度，开展区域性多部门水污染联防联治、水污染防治生态补偿试点。启动总投资26亿元的城区水环境综合整治项目，完成荆襄河湿地公园二期、西干渠智能化远程控制改造等项目，实施护城河及古城内湖泊疏浚清淤、太师渊黑臭水体整治等项目，实现城区水域常态化保洁。推进城市生活污水处理厂扩建。开展农村干渠、河道清淤，集中清理水葫芦、水花生，提高水体自然净化能力。

坚决打好蓝天保卫战。继续抓好“三禁两治”。开展重点企业环境信用评价，建立污染源排放“黑名单”管理制度，严控挥发性有机物污染。推进清洁能源高效集中利用，强化机动车尾气治理和燃油品质管理，大力整治建筑施工、道路、渣土运输等扬尘污染源。实施大气污染防治智能化监管，推进在线监控、精准检测解析，促进空气质量优良天数逐年增加。

加大生态建设力度。实行能源消耗总量和强度“双控”，完成节能减排年度目标任务。扎实开展中央环保督察反馈问题整改“回头看”。继续开展“绿满荆州”行动，加大国家森林城市创建力度，完成植树造林62万亩，森林覆盖率再提高3个百分点。巩固长江禁止非法采砂成果。加快修复洪湖、长湖生态，持续推进退田、退渔、退垸还湖。加快海子湖、菱角湖、崇湖、环荆州古城等国家湿地公园建设。探索滩涂湿地生态保护开发新模式。实施“土十条”，开展全市土壤详查。深入推进农业面源污染治理，支持江陵、监利、石首建设农业面源污染综合治理示范区。开展第二次全国污染源普查和重金属污染详查工作。

（四）构筑综合立体交通。持续加大重大交通项目建设力度，着力破解发展瓶颈。

谋划交通大格局。以“大交通”支撑大城市、推动大发展。积极争取荆荆高铁南延进入规划。谋划推动李埠长江大桥、观音寺长江大桥、监利华容长江大桥等项目。建设车阳河、公安等港区，完善提升盐卡港区功能。大力发展临港产业。谋划航空产业发展，在加快建设荆州民用机场的同时，推进纪南、公安、石首、洪湖等通用机场前期工作。依托机场、铁路、公路、港口、管道，加强多式联运融合发展。

加快重大交通项目建设。全面开工荆州机场航站楼、跑道及相关配套设施建设。加快推进荆荆高铁、江汉平原铁路、蒙华铁路、城北快速路等项目建设。建成高速公路133公里、达到576公里。加快推进洪江高速、沙公高速杨家厂至孟家溪段，石首长江大桥、洪湖嘉鱼长江大桥、洪湖赤壁长江大桥，荆州煤炭铁水联运储配基地一期、长江砂石集并中心等项目建设。启动实施松西河、内荆河航道整治。

提升交通服务保障能力。发挥交通基础设施的先导作用，推动与产业、旅游、扶贫的融合发展。开展普通公路“建养一体化”试点。加快建设“四好农村路”。完成生命安全防护“455”工程3000公里。加快车联网、物联网、大数据、云计算等技术运用，推广无车承运人、甩挂运输等新型运输方式，降低物流成本。实施“公交优先”战略，进一步延伸交通运输服务网络。

（五）推进城乡统筹发展。坚持以人的城镇化为核心，全面提升城乡建设水平，促进城乡一体化发展。

加快建设中心城区。按照“名称不变、体制不变、事权不变、只予不取”原则，积极推动公安、江陵撤县设区。巩固国家卫生城市创建成果，统筹推进全国文明城市、国家森林城市、国家生态园林城市、省级环保模范城市和国家生态文明先行示范区创建。开展新一轮城市总体规划修编。推进城市“双修”。重点抓好中心城区180亿元政府投资重大项目。启动市民中心、市档案馆新馆建设。推进园博园、植物园等项目建设。完成棚户区改造37586套。实施市政截污及污水处理、河道渊湖生态修复等项目。继续推进城区卫生环境整治。新建公共停车场16个，新增停车泊位3800个。实施“还路于民”“亮灯照明”工程。建设50公里城市绿道，打造15分钟健身圈。构建智慧城管、智慧政务、智慧医疗、智慧旅游和智慧教育等平台，打造智慧荆州。

提升县域发展水平。突出特色，走差异化发展之路，促进县域经济对标赶超、争先进位。支持荆州区、沙市区在城市建设、商贸物流、文化旅游等方面发挥引领作用。支持公安县发展装备制造和生物医药产业。支持江陵县发展新型煤电煤化和现代物流产业。支持松滋市启动领军工业企业及税源培植三年行动计划。支持石首市实施“主导产业培育工程”，创建国家生态园林城市。支持监利县撤县设市，完善县城规划、加快城镇化进程。支持洪湖市开展河湖生态修复，做大做强特色旅游。支持卸甲坪乡、川店镇、新沟镇、岑河镇、黄山头镇打造省级特色小镇。

实施乡村振兴战略。按照产业兴旺、生态宜居、乡风文明、治理有效、生活富裕的总要求，制定乡村振兴战略规划，推进“三乡工程”。建设美丽乡村，大力推进“421工程”，实施“厕所革命”，新增50个美丽乡村示范村。继续推进全域旅游，支持松滋市创建国家全域旅游示范区，支持洪湖市创建国家旅游度假区，支持荆州区创建省级全域旅游示范区。加快城乡供气、供水、垃圾处理、污水处理、公共交通一体化进程，促进城乡统筹发展。发挥农民主体作用，广泛利用社会和市场力量，以城带乡、以工促农、城乡融合，撬动和推进农业农村现代化。抓好扶贫创业带头人、农村实用人才、农业科技人才队伍建设，培训新型职业农民3800人。

（六）更加重视改善民生。我们要以造福人民为最大政绩，切实解决好事关人民群众切身利益的突出问题。

持续打好脱贫攻坚战。注重扶贫与扶志、扶智相结合，激发贫困人口内生动力，瞄准特定贫困群体精准帮扶，向深度贫困地区聚焦发力。“因村施计、因户施策”，突出“准、实”导向，下好绣花功夫，集中力量做好产业扶贫、教育扶贫、健康扶贫、金融扶贫和危房改造。因地制宜培育主导产业，完善各类新型经营主体与贫困村、贫困户利益联结机制，带动贫困对象稳定增收。加快实施“百企扶百村”行动，做实做细驻村帮扶工作。强化扶贫资金监管，深入开展扶贫领域不正之风和腐败问题专项整治。完成50个贫困村、46470人脱贫出列任务。

协调发展社会事业。继续推进义务教育均衡发展。优化教育布局，消除“超大班额”，有效缓解“择校热”。改革课堂教学模式，减轻学生课业负担。优化职业教育资源，打造职教特色学校、品牌学科。整合各类资源、资金30亿元，支持长江大学创建“双一流”。支持湖北中医药高等专科学校创建应用型本科院校。支持荆州职业技术学院争创全国优质高职院校。加快长江大学工程技术学院、长江艺术工程职业学院新校区建设。加快“文化三市”创建，创成中国楹联文化城市。举办荆楚文化艺术节。规划建设市文化艺术中心，开工建设公安县、石首市、监利县图书馆和文化馆新馆。改造升级市工人文化宫。建设户外职工“爱心驿站”。实施“一县一团”“戏曲进校园”、艺术人才培养工程。推进健康荆州建设，确保市中心医院、市一医院门诊大楼主体工程完工。创建国家食品安全城市。治理“餐桌污染”，打造一批放心早餐店、夜市示范店。继续推进血吸虫病传播阻断达标工作。全力办好市第五届运动会和第二届荆州国际马拉松。做好第四次全国经济普查相关工作。

加大社会保障力度。实施更加积极的就业扶持政策，统筹解决好高校毕业生、退役军人、城镇困难人员等重点人群就业问题，全年城镇新增就业6.6万人。完善社会保险制度，实施全民参保计划，做到应保尽保。完善城乡最低生活保障制度，统筹扶贫标准和低保标准有效衔接。探索租赁住房建设，保障多样化住房需求。有效遏制房价过快上涨，促进房地产业健康发展。开展智慧养老、社区托老所建设。发展残疾人事业，加强残疾康复服务。支持志愿服务和公益事业发展。

提高社会治理水平。强化公共安全意识，健全安全生产责任制和隐患排查治理体系，深入开展危险化学品、道路交通、建筑施工、消防等行业领域打非治违专项整治。加强和创新社会治理，做好各类风险化解和信访维稳工作，妥善解决群众诉求。扎实开展农民工工资治欠保支工作。推进法治荆州建设，开展基层法治创建活动。推进“平安城市”三期工程建设，开展“一标三实”信息采集，坚决打击“黑枪爆”“盗抢骗”“黄赌毒”，进一步完善立体化、信息化治安防控体系，有效提升人民群众安全感和治安满意度。加强国防教育和后备力量建设，巩固军政军民团结良好局面。充分发挥工会、共青团、妇联等群团组织桥梁纽带作用。继续做好民族宗教、外事侨务、老龄、拥军优属等工作。

三、加强政府自身建设

步入新时代，开启新征程，政府工作必须彰显新气象、展现新作为。我们将认真贯彻落实党的十九大精神，牢记全面建成小康社会重大使命，加强政府自身建设，全力推动经济社会持续健康发展，不辜负党和人民的历史重托。

坚持党的领导，严守政治规矩。坚决维护以习近平同志为核心的党中央权威和集中统一领导，牢固树立“四个意识”。坚决落实省委、省政府和市委的决策部署。深入践行党的群众路线，坚持以人民为中心的发展思想，带着深厚感情和强烈责任为群众办实事、解难题。落实全面从严治党主体责任，加强政府系统党风廉政建设和反腐败斗争。

坚持依法行政，建设法治政府。全面落实法治荆州建设纲要，规范、公正、文明执法，提高政府工作人员依法行政能力和自觉。严格执行重大行政决策法定程序，加强合法性审查。主动接受人大法律监督和工作监督、政协民主监督、司法监督、社会和舆论监督，广泛听取各民主党派和社会各界的意见建议。支持监察体制改革。坚持以公开为常态，不公开为例外，推进政务公开。开展网络问政、电视问政，让政府工作始终置于阳光之下。

坚持担当履职，大兴实干之风。我市正处在发展爬坡期、改革攻坚期、社会转型期，前进道路上面临不少挑战。我们将始终保持昂扬斗志，真正把加快发展的责任扛在肩上，把群众的冷暖忧乐装在心中，困难面前不退缩，矛盾面前不回避，挑战面前不畏惧，主动作为、务实进取，不折不扣完成各项目标。我们将始终坚持真抓实干，为担当者担当，为负责者负责，让想干事、能干事、干成事在全市蔚然成风，推动各项工作干在实处、争先进位。

坚持狠抓落实，提高行政效能。紧紧围绕全市中心工作，紧盯既定目标，以“钉钉子”精神狠抓落实。各项工作安排必须做到时间明确、路径明确、责任明确。进一步完善政府“大督查”体系，推行暗访督查、网上督查、第三方评估，多层次、广渠道、全方位掌握实际情况。严肃督查通报，严格结果考评，抓住不落实的事，处理不作为的人，有责必问、有错必纠，以强有力的督查和问责推动政府每项承诺的兑现。

各位代表！使命光荣艰巨，责任重于泰山。让我们更加紧密地团结在以习近平同志为核心的党中央周围，在中共荆州市委的坚强领导下，在全市人民的支持下，不忘初心、牢记使命，锐意进取、砥砺前行，为“勇当多极排头兵、实现荆州大振兴”作出新的更大成绩！
